# Fine‐scale movements and behaviors of coyotes (*Canis latrans*) during their reproductive period

**DOI:** 10.1002/ece3.7777

**Published:** 2021-06-15

**Authors:** Michael J. Chamberlain, Bradley S. Cohen, Patrick H. Wightman, Emily Rushton, Joseph W. Hinton

**Affiliations:** ^1^ Warnell School of Forestry and Natural Resources University of Georgia Athens GA USA; ^2^ College of Arts and Sciences Tennessee Technological University Cookeville TN USA; ^3^ Georgia Department of Natural Resources – Wildlife Resources Division Social Circle GA USA; ^4^ College of Forest Resources and Environmental Science Michigan Technological University Houghton MI USA

**Keywords:** behavioral state, *Canis latrans*, coyote, movement, resident, space use, transient

## Abstract

In canids, resident breeders hold territories but require different resources than transient individuals (i.e., dispersers), which may result in differential use of space, land cover, and food by residents and transients. In the southeastern United States, coyote (*Canis latrans*) reproduction occurs during spring and is energetically demanding for residents, but transients do not reproduce and therefore can exhibit feeding behaviors with lower energetic rewards. Hence, how coyotes behave in their environment likely differs between resident and transient coyotes. We captured and monitored 36 coyotes in Georgia during 2018–2019 and used data from 11 resident breeders, 12 predispersing residents (i.e., offspring of resident breeders), and 11 transients to determine space use, movements, and relationships between these behaviors and landcover characteristics. Average home range size for resident breeders and predispersing offspring was 20.7 ± 2.5 km² and 50.7 ± 10.0 km², respectively. Average size of transient ranges was 241.4 ± 114.5 km². Daily distance moved was 6.3 ± 3.0 km for resident males, 5.5 ± 2.7 km for resident females, and 6.9 ± 4.2 km for transients. We estimated first‐passage time values to assess the scale at which coyotes respond to their environment, and used behavioral change‐point analysis to determine that coyotes exhibited three behavioral states. We found notable differences between resident and transient coyotes in regard to how landcover characteristics influenced their behavioral states. Resident coyotes tended to select for areas with denser vegetation while resting and foraging, but for areas with less dense vegetation and canopy cover when walking. Transient coyotes selected areas closer to roads and with lower canopy cover while resting, but for areas farther from roads when foraging and walking. Our findings suggest that behaviors of both resident and transient coyotes are influenced by varying landcover characteristics, which could have implications for prey.

## Graphical abstract

We estimated first‐passage time values to assess the scale at which coyotes respond to their environment, and used behavioral change‐point analysis to determine that coyotes exhibited three behavioral states. We found notable differences between resident and transient coyotes in regard to how landcover characteristics influenced behavioral states. Our findings suggest that behaviors of both resident and transient coyotes are influenced by varying landcover characteristics, which could have implications to prey.

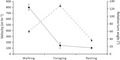

## 1 INTRODUCTION

Space use is an ecological concept that allows us to interpret animal home ranges, habitat selection, foraging strategies, and predator–prey interactions (Moorcroft, 2012; Van Moorter et al., 2016). Animal space use is reflective of an individual's needs and is influenced by resource availability (Jones, 1990; Mills & Knowlton, 1991; Tufto et al., 1996), landscape characteristics (Kittle et al., 2015), predation risk (Tufto et al., 1996), and reproductive needs (Verner, 1977). Predators that are territorial, such as canids, play a substantive role in shaping prey behaviors because predator space use has direct and indirect consequences to prey species that use areas within those territories (Kittle et al., 2015; Krebs, 1980; Tsukada, 1997; Ward et al., 2018). Hence, it is useful to understand predator movements, foraging behaviors, and habitat selection to better understand how they persist in diverse landscapes.

Since the extirpation of red wolves (*Canis rufus*), eastern coyotes (*Canis latrans*) have expanded their range to become the predominant canid predator in the southeastern United States (Hinton et al., 2019; Kilgo et al., 2010). Coyote populations are comprised of resident breeders and their nondispersing offspring that maintain territories and transients (i.e., dispersing and displaced individuals) that exhibit nomadic movements with little fidelity to an area (Gehrt et al., 2009; Gese et al., 1988; Hinton et al., 2015). Because resident coyotes are territorial, understanding their space and habitat use within home ranges can provide relevant information on how they interact with their local environment. Previous studies have reported similarities in space use between resident and transient coyotes, but also have noted that roads can be important to transients (Hinton et al., 2015), similar to other studies noting that canids in general often select roads to move about the landscape (Allen et al., 2013; Baker et al., 2007; Zimmermann et al., 2014). Furthermore, home range size and daily movements of coyotes change temporally depending on reproductive activity and seasonal prey availability (Andelt & Gipson, 1979; Chamberlain et al., 2000; Gosselink et al., 2003). Previous studies have noted that coyotes decrease home range size and daily movements during reproductively active periods (Holzman et al., 1992; Laundré & Keller, 1984), whereas others have found no seasonal variability in home range sizes (Hinton et al., 2015). Coyotes form breeding pairs in which males contribute to pup‐rearing (Bekoff & Wells, 1980), and therefore, little sex‐specific differences in movements and space use exist (Grinder & Krausman, 2001; Laundré & Keller, 1984). However, coyotes select for different habitat types based on seasonal food availability (Mills & Knowlton, 1991) using forested areas in fall and winter but increase the use of early successional habitat and agricultural areas in spring and summer (Hinton et al., 2015; Richer et al., 2001). Coyote space use and movements vary according to individual needs, so understanding fine‐scale space use and movement behaviors is important to understanding interactions between coyotes and their environment.

Broad patterns of coyote space use are well studied, but fine‐scale movements and behaviors during their reproductive seasons remain poorly understood. Coyotes are reproductively active from February until June (Gier, 1968) in which they may exhibit different foraging strategies relative to other periods of the year because adults need to provide their pups food and protection (Andelt & Gipson, 1979; Harrison & Harrison, 1984; Messier & Barrette, 1982). Predators encountering potential prey often adjust movement patterns accordingly (Fauchald, 1999), exhibiting area‐restricted searching (Kareiva & Odell, 1987). Time spent in area‐restricted searching can reflect foraging behaviors, and the spatial scale associated with such behaviors can be quantified using first‐passage time analysis (Fauchald & Tveraa, 2003). Coyotes exhibit behavioral responses to different environmental factors and analysis of movement data can provide insights into how they exploit resources at various spatial scales (Gurarie et al., 2009; Schick et al., 2008), especially during their reproductive seasons. Our objectives were to describe fine‐scale patterns of space use and detail daily movements of coyotes during the spring and summer when coyotes are reproductively active. We also sought to detail how landscape characteristics influenced coyote movement behaviors. We broadly hypothesized that space use and movements would differ between resident and transient coyotes during the reproductive season and that some landscape characteristics would influence coyote movement behaviors. We predicted that coyote space use and movement would increase linearly from spring when pups are whelped and dependent on milk for nutrition (restricted movements for adults) to summer when pups were no longer dependent on milk and could freely move on their own (less restricted movements for adults). We predicted that denser vegetation and forest edges would be associated with foraging behaviors, whereas roads and areas with less dense vegetation would be associated with movements across the landscape.

## 2 STUDY AREA

**FIGURE 1:**
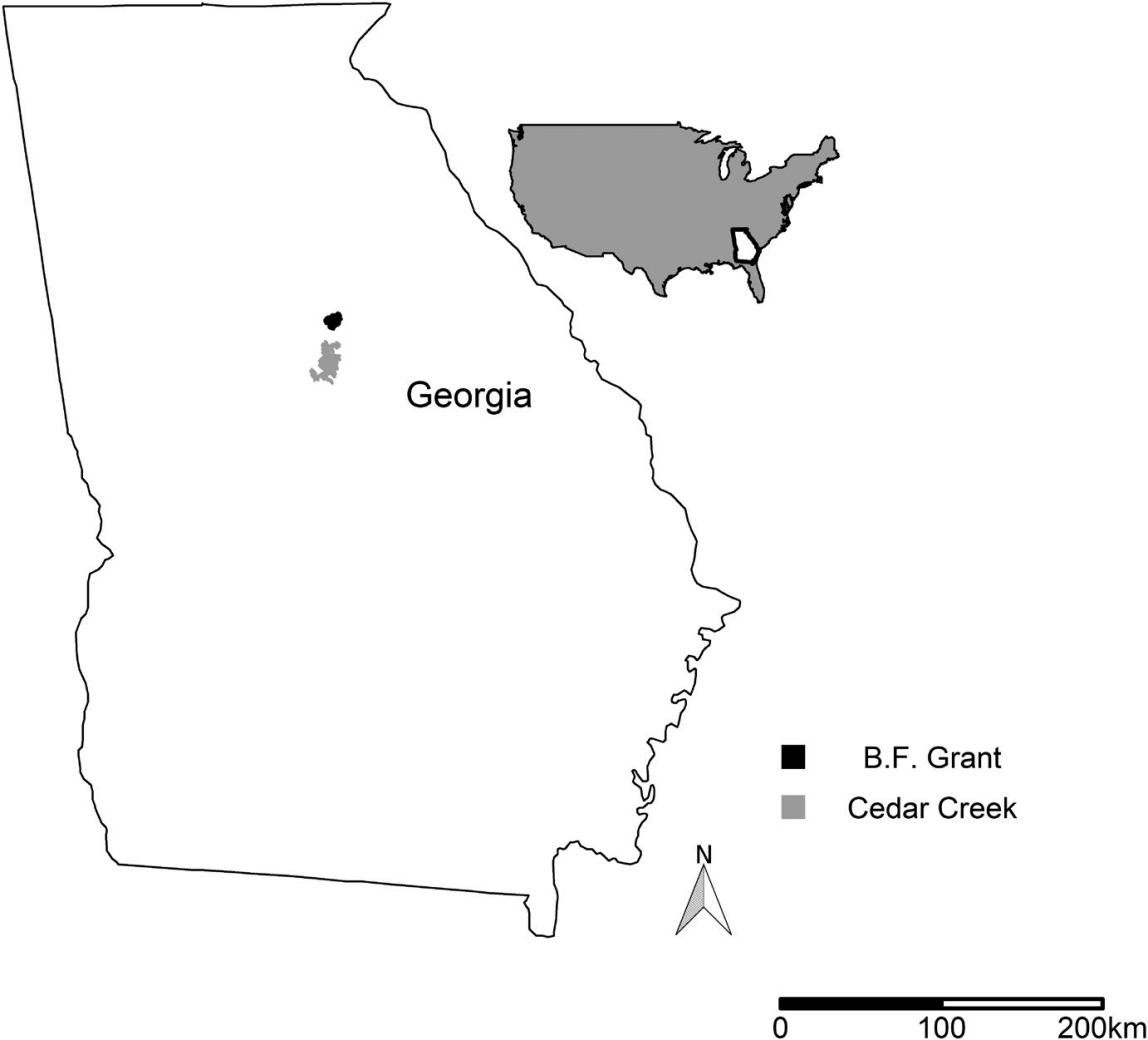
B.F. Grant and Cedar Creek Wildlife Management Areas subset in a map of Georgia, USA

We conducted research in the Piedmont region of central Georgia on Cedar Creek and B. F. Grant Wildlife Management Areas (WMA) and surrounding privately owned property (Figure 1). B. F. Grant was a 4,613‐ha area located in Putnam and Morgan County owned by the Warnell School of Forestry and Natural Resources at the University of Georgia and managed by the Georgia Department of Natural Resources Wildlife Resources Division (GADNR). Contained within B. F. Grant WMA was the University of Georgia Beef Unit, which included a mosaic of agricultural fields. Agricultural areas were mostly grazed mixed fescue (*Festuca* spp.) fields and hay fields planted in rye grass (*Lolium* spp.). Forested areas consisted of loblolly pine (*Pinus taeda*) forests, mixed hardwood and pine forests, and hardwood lowlands containing white oak (*Quercus alba*), sweetgum (*Liquidambar styraciflua*), yellow poplar (*Liriodendron tulipifera*), hickory (*Carya* spp.), and other oak species (*Quercus* spp.). The understory was dominated by sweetgum, eastern redbud (*Cercis canadensis*), muscadine (*Vitis rotundifolia*,), flowering dogwoods (*Cornus florida*), and briars (*Rubus* spp.). Forests on B. F. Grant were managed with patch cuts, thinning, and prescribed fire during the dormant season (January–March). Private lands surrounding the WMA were primarily managed for timber production or agricultural practices.

Cedar Creek WMA was a 15,303‐ha area located in Putnam, Jasper, and Jones counties, owned by the U.S. Forest Service (USFS) and managed collectively with GADNR. The site was comprised of managed loblolly pine forests, mixed hardwood and pine forests, hardwood valleys containing mostly white oak, sweetgum, yellow poplar, hickory (*Carya* spp.), and other oak species, and small openings for wildlife. The understory was similar to that on B. F. Grant WMA. Cedar Creek WMA forests were managed by timber thinning and periodic dormant season fire. Private lands surrounding the WMA were managed primarily for timber production or agricultural practices. Both study sites and surrounding private lands had an open hunting season on coyotes year‐round, but the amount of hunting pressure on coyotes and harvest were unknown.

## 3 METHODS

Coyotes were captured by a professional trapper using MB‐450 foothold traps (Minnesota Brand traps, Minnesota Trapline Products, Inc.) during January–March 2018–2019. Coyotes were restrained using a catchpole, muzzle, and hobbles, then weighed, sexed, and age was estimated based on tooth wear and body condition (Gier, 1968; Gipson et al., 2000). We categorized coyotes ≥2 years old as adults and ≤2 as juveniles. Before release, coyotes were fitted with Iridium GPS collars (Advanced Telemetry Systems G5‐2A GPS‐Iridium, Lotek LiteTrack Iridium 360). To maximize battery life, we programmed the collars to record two locations a day at 0000 and 1200 from the date of capture until 1 March. We considered 1 March the beginning of the pup‐rearing period and began an intensive tracking schedule of 1 location every hour until July 31.

Following Hinton et al. (2015), we considered that coyotes only exhibited two disparate space use strategies in which coyotes participated in defending territories as either resident breeders or predispersing offspring, or they were solitary transients nomadically traversing the landscape searching for mates and vacant ranges. Resident coyotes were comprised of breeding pairs and their predispersing offspring that defended territories, whereas transient coyotes were solitary individuals that traverse greater distances than residents did and did not exhibit fidelity to a specific area (Gese, 2001; Hinton et al., 2015; Kamler & Gipson, 2000). We distinguished resident and transient individuals based on their ability to occupy a stable and defined home range, and on whether individuals exhibited stable weekly space use throughout the reproductive period. However, nondispersing offspring can exhibit more plasticity in movements than breeders as they exhibit predispersal movements to ultimately leave their natal home ranges. Hence, we separated individuals into three social classes (resident breeder, predisperser, transient) but retained two space use strategies for subsequent analyses. For example, when estimating weekly space use and daily distances traveled, we assessed all three social classes, but then only considered resident and transient space use strategies when investigating movement behaviors and habitat selection. Predispersers exhibit similar and overlapping use of their pack's territory while making excursions that display transient‐like movements for short durations. Eventually, predispersers permanently leave their natal territories and enter transiency until they establish residency with a mate or die trying (Hinton et al., 2015). Therefore, because most predisperser movements were within their natal territories, we considered them residents when assessing movement behaviors and habitat selection.

Following Hinton et al. (2015), we estimated space use of residents, predispersers, and transient coyotes with dynamic Brownian bridge movement models (dBBMMs) fit to time‐specific location data, using package move (Kranstauber et al., 2012, 2019) in Program R (R Core Team, 2020) with a margin size of 3, window size of 15, and location error of 20 m. We calculated 50% core use and 99% home range areas for each individual from 1 March–31 July, and then weekly during this period to assess potential changes in space use throughout the reproductive season. Due to missing fixes and collar malfunction, when constructing weekly ranges, we only used individuals where >50 locations were collected during the week with ≥2 locations daily. Because transients did not maintain territories, we considered 99% and 50% contour intervals as transient ranges and biding areas, respectively (Hinton et al., 2012, 2015). As noted by Hinton et al. (2015), biding areas are areas that experience localized use by transient coyotes and may represent attempts by transients to establish territories (settlement).

We estimated mean daily distance moved for each coyote by summing the sequential distances between GPS locations in a 24‐hr period using program R (R Core Team, 2020). We calculated an average daily distance moved for the entire period that each coyote was monitored, and also calculated average daily distance moved per week. We used *t* tests with an *α* = 0.05 to investigate differences in space use and daily distance metrics between sexes within social units. We used a Shapiro–Wilk test to evaluate assumptions of normality and applied transformations when necessary to ensure data were normally distributed. We used a Levene's test to test assumptions of homogeneity of variance.

We examined temporal trends in weekly home ranges and core areas for resident breeders and predispering offspring and transient ranges and biding areas for transients as well as average daily distance traveled per week via generalized additive mixed models (GAMM) using R package mgcv (Wood, 2006). We used week as a predictor variable and weekly space use and average daily distance traveled as response variables. We visually evaluated results based on predicted effect sizes and confidence limits. We fitted week as a spline variable and considered individual coyotes nested within packs as random effects. Because transient coyotes were solitary individuals unassociated with packs, we only included individual coyotes as the random effect. We fit separate models for resident breeders, predispersers, and transients.

### 3.1 First‐passage time analysis

We estimated first‐passage time (FPT) values to quantify the spatial scale at which resident and transient coyotes responded to their environment (Bissonette, 2017). Byrne et al. (2014) and Cohen et al. (2019) both recently used FPT values to infer the scale at which wild turkeys (*Meleagris gallopavo*) reacted to changes within their environment. We used similar methodology to infer the scale at which coyotes responded to their environment. We calculated FPT values for circles with radius *r* ranging from 10 to 400 m in 10 m increments along individual movement paths. For each movement path, we extracted the value of *r* with the greatest variance in log transformed FPT values and determined this as the scale at which a coyote concentrated its activities (Fauchald & Tveraa, 2003). Scale varied across different individual paths; therefore, we used the largest mean variance (164.7 m) averaged across all paths to define a common scale for analysis. At this scale, we assumed that behavioral responses were influenced by environmental stimuli. We quantified covariates associated with landcover classifications (described below) by calculating mean distance to landcover values within a 164.7 m radius buffered circle around each hourly GPS location.

### 3.2 Landcover classification

We used a combination of several spatial layers to describe various landcover and landscape characteristics, and then model their influences on coyote behaviors. Specifically, we sought metrics for vegetation density, forest canopy cover, distance to landcover types known to influence coyote behaviors during spring and summer (Hinton et al., 2015; Tigas et al., 2002), and distance to forest edges and roads, which are known to influence behaviors of canids (Dellinger et al., 2013). We used the National Land Cover Database (NLCD, Homer et al., 2015) and normalized difference vegetation index (NDVI) to determine landcover types and vegetation density (Pettorelli et al., 2005), respectively. We determined canopy cover using the NLCD tree canopy cover layer (Homer et al., 2015). We delineated riparian cover using the NLCD layer by including landcover types categorized as both woody wetlands and emergent herbaceous wetlands. Similarly, we delineated forest edge using the NLCD layer by including landcover types categorized as hardwood forest, conifer forest, and mixed conifer/hardwood forest and by extracting linear edges of those forest types. We identified roads using 2019 USGS Tiger/Line data (Topologically Integrated Geographic Encoding and Referencing) and considered this landscape characteristic as our representative of anthropogenic land cover. We then used the Euclidean Distance tool in the Spatial Analyst toolbox in ArcMap 10.7 (Environmental Systems Research Institute) to create distance raster maps for all landcover types and calculated the distance from each pixel to each landcover type and road (Benson, 2013).

### 3.3 Behavioral state analysis

Accurately defining behaviors and understanding what environmental factors influence certain behaviors provide information on how animals use space. Therefore, we quantified coyote behavior in different landcover classes and evaluated how landcover classes were related to various movement behaviors. We used behavioral change‐point analysis (BCPA) to model coyote behavior (Gurarie et al., 2009) using methods outlined in Cohen et al. (2019). The BCPA is a likelihood‐based method that identifies changes in movement patterns using locational time‐series data. The BCPA detects changes in movement through velocities and relative turn angles (RTAs) using a sweeping window analysis and temporal autocorrelation associated with GPS locations. The BCPA then estimates the most likely location where a change in movement parameters occurred according to a Bayesian Inference Criterion (BIC; Gurarie et al., 2009). We used BCPA to interpret movement behaviors because it can reveal behavioral states in GPS tracking data without prior assumptions regarding the distributions of movement parameters (Gurarie et al., 2009, 2016).

We calculated velocity and RTA between all GPS locations along a path for each individual coyote. We used a smoothed BCPA analysis in package “bcpa” (Gurarie, 2014), and a window size of 30 sequential locations and sensitivity (*K*) of 1 (Gurarie et al., 2016). This approach allowed us to identify changes in behavioral states at the smallest temporal scale while still meeting the minimum sample size required for the BIC model selection (Gurarie et al., 2009). We hereafter refer to segments of paths between change points (i.e., locations where shifts in movement parameters occurred) as bouts. We calculated median values for these metrics because the distribution of velocity and RTA values were positively skewed.

We determined the number of distinct behavioral states for each coyote using a hierarchical clustering method to assess within‐group sum of squares and classification of bouts (Krzanowski & Lai, 1988) based on velocity and RTA combinations (Zhang et al., 2015). We used *k‐*means clustering (Hartigan & Wong, 1979) in package “cluster” (Maechler et al., 2017) and “fpc” (Hennig, 2018) to classify movement bouts into different behavioral states. Unique bouts, identified by the BCPA, allowed us to categorize behavioral states according to similar movement patterns. We chose to limit our *k‐*means clustering to three behavioral states (Zhang et al., 2015): foraging, walking, and resting. Increased RTA movements with lower velocities indicated area‐restricted search behaviors, which presumably meant a coyote was searching and foraging (Gurarie et al., 2016). The walking state showed directional movement with lower RTA and greater velocities (Gurarie et al., 2016). Resting behaviors were characterized by lower RTA and reduced velocities (Gurarie et al., 2016; Zhang et al., 2015). We then assigned each GPS location to a behavioral state.

We were interested in which behavioral states were induced by NDVI, percent canopy cover, and distances to forest edge, riparian cover, and roads. To distinguish these differences, we calculated the number of locations spent in each behavioral state and then calculated the mean and standard error within each behavioral state across individuals. We then described behavior within each landcover type by calculating the number of locations in each behavioral state for every coyote in each landcover type. Lastly, we calculated the mean and 95% confidence interval of these proportions across coyotes.

We conducted generalized additive mixed models (GAMM) using package mgcv (Wood, 2006) to examine the explanatory power of proximity to landcover types and roads on coyote behaviors. We included random intercepts for each coyote (Coyote ID) nested within pack (Pack) in models for resident coyotes. Including random intercepts for individual coyotes and pack identity accounted for influences of unmeasured individual‐ and pack‐related factors on behavioral states, given the clustered nature of the data. Because transient coyotes were solitary individuals unassociated with packs, we only included Coyote ID as a random effect to account for individual variability. We determined statistical significance at *α* = 0.10. We modeled distance to different landcover types and roads by analyzing three separate GAMMs for both resident and transient coyotes with a binary response variable wherein we assigned the behavioral state of interest (e.g., walking) as 1 and the other behavioral states (e.g., foraging and resting) as 0. Before modeling, we rescaled and centered values for distance‐based variables, NDVI, and canopy cover by subtracting their mean and dividing by 1 *SD*. We then explored all possible subsets of the five predictors as candidate models to investigate each of the three behavioral states for resident and coyotes. We evaluated model sets using Akaike's information criterion, adjusted for small sample sizes (AIC_c_), and used ΔAIC_c_ to select which models best‐supported predictors influencing resource selection during each behavioral state exhibited by coyotes (Burnham & Anderson, 2002).

## 4 RESULTS

We caught 36 coyotes (16 M, 22 F) during 2018 and 2019; due to early transmitter malfunctions and mortality, we used GPS data from 33 individuals to determine ranges and daily movements. We classified 11 individuals as transients (5 M, 6 F), 10 as residents (4 M, 6 F), and 12 as predispersers (6 M, 6 F). Average home range (20.72 ± 2.49 km², mean ± *SE*) and core area sizes (1.44 ± 0.19 km^2^) for the entire study period did not differ by sex for residents (*t*_5_ = −0.94, *p* = 0.39, and *t*_5_ = −0.23, *p* = 0.39, respectively). Likewise, we detected no difference by sex in average home range (50.70 ± 10.00 km²; *t*_4_ = 0.02, *p* = 0.98) and core area sizes (2.40 ± 0.57 km^2^; *t*_4_ = 0.25, *p* = 0.81) for predispersers. We also detected no difference by sex in average transient range (241.44 ± 114.53 km^2^; *t*_5_ = −0.63, *p* = 0.55) and biding area sizes (7.30 ± 2.22 km^2^; *t*_5_ = 0.61, *p* = 0.57) for transients.

**FIGURE 2:**
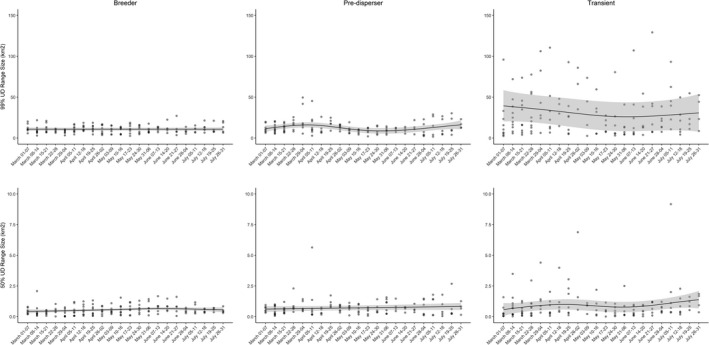
Generalized additive regression predictions (95% confidence intervals in gray) for estimated weekly 50% and 99% utilization distributions from 33 coyotes (*Canis latrans*) classified as resident breeders, predispersers, and transients on Cedar Creek and B. F. Grant Wildlife Management Areas (WMA), Georgia, USA, and surrounding privately owned properties between 1 March and 31 July during 2018–2019

We detected little variation in weekly range and core area sizes for residents, predispersers, and transients (Figure 2). We noted the general trend that space use varied little across weeks for residents, more so for predispersers, and the most for transients. We observed no difference in weekly home range (10.63 ± 0.4 km^2^; *t*_148_ = −1.53, *p* = 0.13) and core area sizes (0.53 ± 0.03 km^2^; *t*_148_ = −0.02, *p* = 0.98) between male and female residents, nor did weekly transient range (32.2 ± 2.02 km^2^; *t*_135_ =0.95, *p* = 0.35) or biding area sizes (0.86 ± 0.1 km^2^; *t*_135_ = 1.64, *p* = 0.10) differ by sex for transients. However, predispersing males maintained larger weekly home ranges (14.6 ± 1.0 km^2^; *t*_135_ = −2.36, *p* = 0.02) and core areas (0.90 km^2^ ± 0.1; *t*_135_ = −3.22, *p* = 0.001) compared with predispersing females (range = 11.5 ± 0.8 km^2^, core area = 0.57 ± 0.04 km^2^, Supporting information).

**FIGURE 3:**
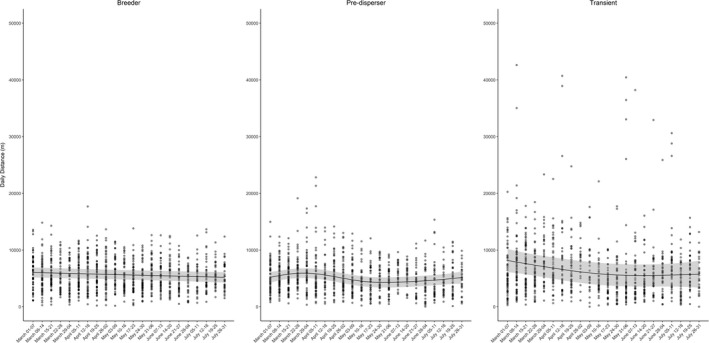
Generalized additive regression predictions (95% Confidence Interval in gray) for average daily distance traveled per week for 33 coyotes (*Canis latrans*) on Cedar Creek and B. F. Grant Wildlife Management Areas (WMA), Georgia, USA, and surrounding privately owned properties between 1 March and 31 July during 2018–2019

We noted that daily distances traveled across weeks followed trends in weekly space use, with little variation in distances traveled by residents and more variation for predispersers and transients (Figure 3). The average daily distance traveled for all coyotes during the entire study period was 5.8 ± 0.08 km (mean ± *SE*), with the trend being that transients moved farther per day than residents (Table 1). We found that average daily distance moved across weeks was greater for males than females in all social units (Appendix S1).

**TABLE 1:** Mean daily distance moved (m) and associated standard errors (*SE*) by resident breeder, predisperser, and transient male and female coyotes (*Canis latrans*) from 1 March to 31 July in the Piedmont region of Georgia during 2018–2019

Social unit	Sex	*n* ^a^	Daily Distance	*SE*	*t*	*df*	*p*‐value
Resident breeder	Female	6	5,437	107.76	−3.80	990	<0.05
	Male	4	6,137	141.66			
Predisperser	Female	6	4,524	109.03	−10.96	926	<0.05
	Male	6	6,412	142.29			
Transient	Female	6	5,821	155.28	−6.53	906	<0.05
	Male	5	8,009	304.34			

Note

We present results from a 2‐sample *t* test evaluating differences between daily distance traveled by males and females within social units.

^a^ Number of coyotes monitored within each type of social unit.

**FIGURE 4:**
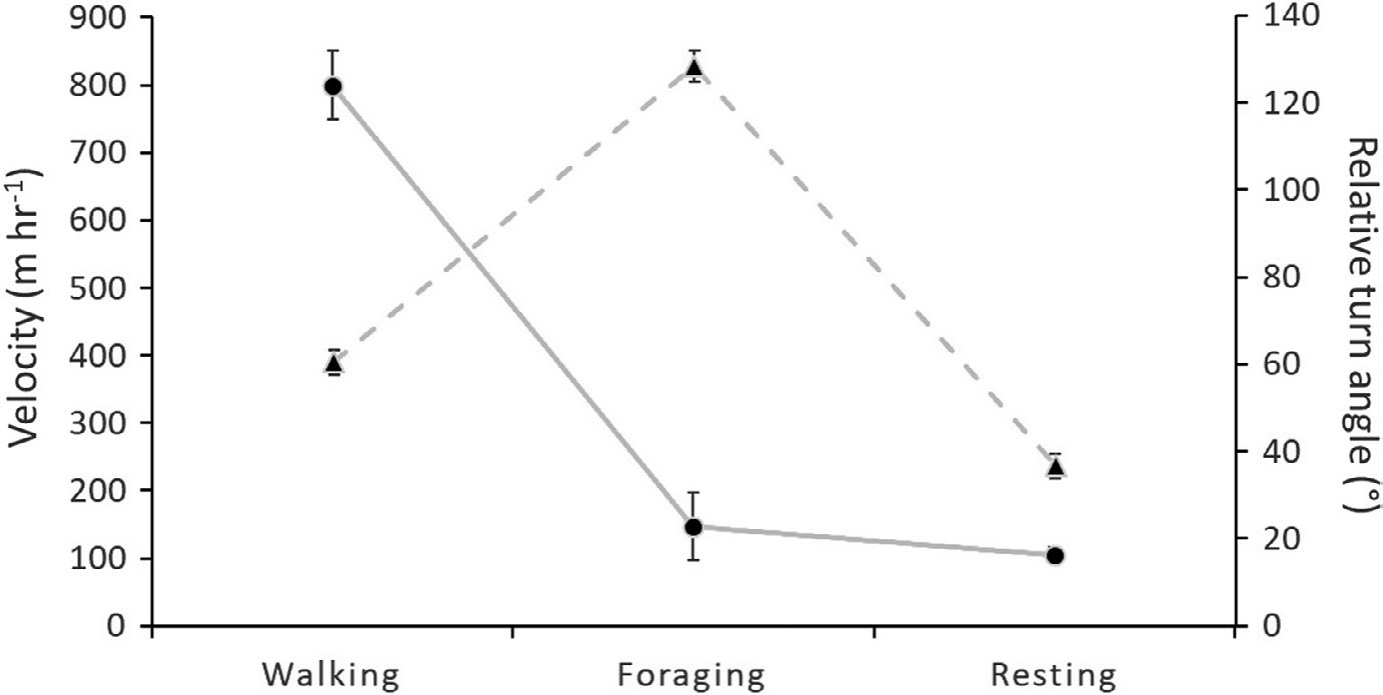
Statistical definitions of behavioral states inferred for GPS locations of 33 coyotes (*Canis latrans*) monitored on Cedar Creek and B. F. Grant Wildlife Management Areas (WMA), Georgia, USA and surrounding privately owned properties during 1 March to 31 July, 2018–2019. Behaviors were classified by sequential use of behavioral change‐point and *k*‐means clustering analyses, based on combinations of between‐relocation velocities (solid line) and relative turn angles (dotted line). Dots represent mean values, and vertical bars represent 95% confidence intervals

Resident coyotes exhibited 3 distinct behavioral states along their movement paths (Figure 4). One behavioral state was characterized by slow velocity (median velocity = 105.9 ± 10.9 m/hr) and low turning angles (median RTA = 36.7 ± 2.9°). We considered this behavior as resting and assumed individuals in this state were likely loafing at den or rendezvous sites. A second behavioral state was considered walking, characterized by relatively fast (median velocity = 799.4 ± 50.5 m/hr) and comparatively straight (median RTA = 60.4 ± 2.9°) movement paths. The third behavioral state had lower velocity (median velocity = 147.3 ± 50.0 m/hr) but greater mean turn angles (median RTA = 128.2 ± 3.6°). We interpreted this as area‐restricted search behavior and classified this state as foraging. Time spent in each behavioral state varied across individual coyotes, but on average resting accounted for 62.2 ± 1.3% (range 41.9%–88.4%), walking 8.6 ± 0.7% (range: 0.2%–17.6%), and foraging for 28.1 ± 1.1% (range: 4.2%–45.2%) of individual locations.

As expected, GAMMs indicated that behavioral states were influenced by landcover types, roads, and space use status (resident vs. transient coyotes; Table 2). While resting, residents were more likely to select areas farther away from roads and riparian cover, and in areas with denser vegetation (Table 3, see Appendix S1). When foraging, residents were closer to riparian areas while also selecting for denser vegetation (Table 3, see Appendix S1). While walking, residents selected for roads and riparian areas, and with less dense vegetation and canopy cover (Table 3, see Appendix S1). Transient coyotes selected for areas closer to roads and with less canopy cover while resting (Table 4, see Appendix S1). When foraging, transients selected areas closer to forest edges and farther from roads, and in areas with denser canopy cover. Transients selected for areas farther from roads and with less canopy cover while walking (Table 4, see Appendix S1).

**TABLE 2:** Summary of the top five generalized additive mixed models for explaining habitat selection by resident and transient coyotes during three behavioral states in the Piedmont region of Georgia, 2018–2019

Coyote status	Behavioral state	Model	*K*	Deviance	ΔAIC_c_	*ω_i_*
Resident	Resting	NDVI + riparian + roads	6	−17,868.85	0.00	0.48
		Forest edge + NDVI + riparian + roads	7	−17,868.68	1.67	0.21
		Canopy cover + NDVI + riparian + roads	7	−17,868.77	1.84	0.19
		Canopy cover + forest edge + NDVI + riparian + roads	8	−17,868.56	3.41	0.09
		Riparian + roads	5	−17,873.54	7.39	0.01
	Walking	Canopy cover + NDVI + riparian + roads	7	−8,078.03	0.00	0.50
		Canopy cover + forest edge + NDVI + riparian + roads	8	−8,077.37	0.68	0.36
		Forest edge + NDVI + riparian + roads	7	−8,080.29	4.53	0.05
		NDVI + riparian + roads	6	−8,081.45	4.84	0.04
		Canopy cover + NDVI + roads	6	−8,082.30	6.55	0.02
	Foraging	Canopy cover + NDVI + riparian	6	−16,125.33	0.00	0.30
		Canopy cover + forest edge + NDVI + riparian	7	−16,125.29	1.94	0.12
		Canopy cover + NDVI + riparian + roads	7	−16,125.30	1.96	0.11
		NDVI + riparian	5	−16,127.49	2.33	0.09
		Canopy cover + riparian	5	−16,127.52	2.38	0.09
Transient	Resting	Canopy cover + forest edge + roads	5	−7,905.21	0.00	0.16
		Canopy cover + roads	4	−7,906.55	0.68	0.11
		Roads	3	−7,907.62	0.82	0.11
		Forest edge + roads	4	−7,906.94	1.47	0.08
		Canopy cover + forest edge + riparian + roads	6	−7,908.10	1.80	0.07
	Walking	Canopy cover + forest edge + roads	5	−3,036.17	0.00	0.15
		Canopy cover + forest edge + NDVI + roads	6	−3,035.35	0.36	0.13
		Canopy cover + forest edge	4	−3,037.47	0.60	0.11
		Canopy cover + roads	4	−3,037.49	0.63	0.11
		Canopy cover + NDVI + roads	5	−3,036.50	0.66	0.11
	Foraging	Canopy cover + forest edge + roads	5	−7,203.21	0.00	0.27
		Canopy cover + forest edge + NDVI + roads	6	−7,202.72	1.03	0.16
		Canopy cover + forest edge	4	−7,204.83	1.23	0.15
		Canopy cover + forest edge + riparian + roads	6	−7,203.21	1.99	0.10
		Canopy cover + forest edge + NDVI	5	−7,204.45	2.48	0.08

Note

Shown are number of parameters (*K*), Akaike's information criteria for small sample sizes (AIC_c_), differences among AIC_c_ (ΔAIC_c_), and AIC_c_ weights (*ω_i_*).

**TABLE 3:** Parameter estimates (*β*; logit scale) with associated standard errors (*SE*), *Z* values, and *p*‐values for behavioral state models examining how distance to landcover types induced behaviors (resting, walking, foraging) of resident (breeders and predispersers) coyotes in the Piedmont region of Georgia during 2018–2019

Behavioral state model	Covariate	*β* ^a^	*SE*	*Z*	*p*
Resting					
	Intercept	0.524	0.058	9.002	<0.01
	Distance to forest edge	0.010	0.016	0.652	0.51
	Distance to riparian	0.061	0.014	4.265	<0.01
	Distance to roads	0.049	0.016	3.064	<0.01
	Percent canopy	−0.008	0.015	−0.507	<0.01
	NDVI	0.068	0.018	3.813	<0.01
	Pack^b^	0.000	NA	NA	NA
	Coyote ID^c^	0.067	NA	NA	NA
Walking	Intercept	−2.421	0.109	−22.203	<0.01
	Distance to forest edge	−0.030	0.026	−1.144	0.25
	Distance to riparian	−0.069	0.024	−2.862	<0.01
	Distance to roads	−0.121	0.029	−4.582	<0.01
	Percent canopy	−0.059	0.024	−2.437	0.02
	NDVI	−0.300	0.029	−10.396	<0.01
	Pack^b^	0.027	NA	NA	NA
	Coyote ID^c^	0.124	NA	NA	NA
Foraging					
	Intercept	−0.929	0.065	−14.298	<0.01
	Distance to forest edge	−0.004	0.017	−0.249	0.80
	Distance to riparian	−0.041	0.015	−2.668	<0.01
	Distance to roads	−0.004	0.017	−0.211	0.84
	Percent canopy	0.034	0.016	2.094	0.04
	NDVI	0.039	0.017	2.068	0.04
	Pack^b^	0.001	NA	NA	NA
	Coyote ID^c^	0.008	NA	NA	NA

^a^ Variable are scaled to aid in model convergence. Distance to habitat types was divided by 200. Parameter estimate on logit scale.

^b^ Pack was considered to be a random effect in the model. Thus, it is an estimate of standard deviation of the random effect term.

^c^ Coyote ID was considered to be a random effect in the model. Thus, it is an estimate of standard deviation of the random effect term.

**TABLE 4:** Parameter estimates (*β*; logit scale) with associated standard errors (*SE*), *Z* values, and *p*‐values for behavioral state models examining how distance to landcover types induced behaviors (resting, walking, foraging) of transient coyotes in the Piedmont region of Georgia during 2018–2019

Behavioral state model	Covariate	*β* ^a^	*SE*	*Z*	*p*
Resting					
	Intercept	0.584	0.058	10.040	<0.01
	Distance to forest edge	0.035	0.021	1.619	0.11
	Distance to riparian	−0.010	0.022	−0.442	0.66
	Distance to roads	−0.055	0.021	−2.619	<0.01
	Percent canopy	−0.043	0.023	−1.862	0.06
	NDVI	−0.006	0.041	−0.157	0.88
	Coyote ID^b^	0.026	NA	NA	NA
Walking					
	Intercept	−2.752	0.489	−5.624	<0.01
	Distance to forest edge	0.056	0.037	1.527	0.13
	Distance to riparian	−0.004	0.038	−0.100	<0.920
	Distance to roads	0.070	0.039	1.803	0.07
	Percent canopy	−0.237	0.040	−5.954	<0.01
	NDVI	−0.212	0.165	−1.285	<0.20
	Coyote ID^b^	2.119	NA	NA	NA
Foraging					
	Intercept	−0.962	0.050	−19.266	<0.01
	Distance to forest edge	−0.062	0.023	−2.704	<0.01
	Distance to riparian	0.000	0.023	0.006	0.99
	Distance to roads	0.041	0.022	1.845	0.07
	Percent canopy	0.124	0.025	4.977	<0.01
	NDVI	−0.037	0.038	−0.988	0.32
	Coyote ID^b^	0.017	NA	NA	NA

^a^ Variable are scaled to aid in model convergence. Distance to habitat types was divided by 200. Parameter estimate on logit scale.

^b^ Coyote ID was considered to be a random effect in the model. Thus, it is an estimate of standard deviation of the random effect term.

## 5 DISCUSSION

Coyote space use has been extensively studied across the southeastern United States, but previous works focused on coarse‐scale evaluations of seasonal patterns in space use. To our knowledge, our study is the first to combine high‐resolution spatial data with landscape characteristics to assess fine‐scale movements and space use of both resident and transient coyotes throughout the pup‐rearing season. We found that resident coyotes had home range and core area sizes comparable to those reported in contemporary studies (Hickman et al., 2016; Mastro et al., 2019; Ward et al., 2018) and noted a similar trend in regard to daily movements (Grubbs & Krausman, 2009). It was not surprising that we observed comparable movements and space use with previous studies in the southeastern United States, given the relative consistency of body sizes exhibited by coyotes throughout much of the region (Hinton et al., 2019). Conversely, our findings relative to landscape characteristics associated with various behavioral states offer unique information about coyote movement ecology. Our findings supported our hypothesis that some landscape characteristics would influence coyote movement behaviors. Specifically, we observed that coyotes foraged in areas with increasing vegetation density and closer to riparian areas, whereas they rested/loafed in areas with denser vegetation farther from roads. Likewise, we observed that coyotes moving about their ranges (walking) avoided denser vegetation and used areas near roads. Collectively, our findings provide a detailed assessment of how coyotes behave on the landscape and offer relevant information about coyote movements and how movements may relate to coyote use of land cover.

Coyote space use is important for reproductive success, individual survival, and maintenance (Verner, 1977). Despite different kinds of technology (i.e., VHF versus GPS) and home range estimators used to quantify coyote space use, mean home range sizes recorded for coyotes across North America are typically <70 km² (Hinton et al., 2015) and Ward et al. (2018) found that coyote home ranges across broad areas of the southeastern United States varied from 2.8 to 72.9 km², with resident individuals averaging 17.6 km². Across our study period (March–July), we observed that resident breeders maintained home ranges of approximately 21 km^2^, whereas on a weekly basis these same ranges averaged ~10 km^2^. We also noted that resident predispersers maintained weekly ranges and core areas comparable to those of resident breeders, although predispersers are known to make excursions from natal ranges while resident breeders typically do not (Kamler & Gipson, 2000). Conversely, transients maintained markedly larger ranges and biding areas, both on a weekly basis and across our study period, commensurate with the more variable and unpredictable movements associated with transiency (Chamberlain et al., 2000; Hinton et al., 2012, 2015).

Animal movements are reflective of their responses to biotic and abiotic conditions within their ranges (Svoboda et al., 2019). We observed that on average, coyotes moved ~6 km per day within their home ranges, comparable to more contemporary studies detailing coyote movements using GPS telemetry (Grubbs & Krausman, 2009). We also noted that daily movements tended to show similar trends as weekly space use, in that coyote movements remained stable throughout the pup‐rearing season for both classes of residents but were more variable for transients. Increased variability observed in transient movements may have resulted from extensive movements associated with long‐distance dispersal (Hinton et al., 2012) and shifting space use (Hinton et al., 2015). Previous studies have reported temporal and spatially consistent patterns in both space use and movements of resident coyotes throughout their annual cycle (Gifford et al., 2017; Hinton et al., 2015; Young et al., 2006). Conversely, transiency is characterized by nomadic movements where transient individuals may avoid residents while also searching the landscape for vacant territories (Hinton et al., 2015; Morin & Kelly, 2017). We suspect that the relative stability in space use and movements by resident coyotes during the pup‐rearing season is likely influenced by their wide diet breadth and inextricable link to early successional vegetation communities for foraging (Stevenson et al., 2019; Ward et al., 2018).

Habitat selection by coyotes has been extensively studied, with most authors noting that coyotes select for agriculture and other early successional vegetation communities (Hinton et al., 2015; Kamler & Gipson, 2000; Schrecengost et al., 2008; Ward et al., 2018). We linked patterns of habitat selection to inferred behavioral states, thereby offering the opportunity to assess how land cover characteristics influenced coyote movement behaviors. We observed that coyotes spent 62% of their time during the pup‐rearing season in a resting state, presumably while inactive and loafing at rendezvous points, day beds, and den sites (Andelt, 1985; Harrison & Gilbert, 1985; Way et al., 2001). For resident coyotes, these inactive periods are spent nursing and caring for pups, and as pups age, they also explore areas around dens and rendezvous sites as attending adults show them areas within their natal ranges (Harrison & Gilbert, 1985; Messier & Barrette, 1982). We observed that resident coyotes spent time resting farther from roads and riparian areas, and in areas with denser vegetation. Conversely, we noted that transient coyotes tended to rest closer to roads in areas with less forest canopy cover. Previous authors noted avoidance of roads by resident coyotes throughout broad areas of the southeastern United States (Hinton et al., 2015; Ward et al., 2018), and transient coyotes may exhibit stronger selection for roads than residents (Hinton et al., 2015).

Presumably, walking behaviors were associated with coyotes moving rapidly along travel routes, likely traversing through unprofitable areas to reach quality‐foraging sites or attempting to intercept or chase larger prey (Lingle, 2001; Royama, 1970). We found that resident coyotes tended to exhibit walking behaviors closer to roads in areas with reduced vegetation density and forest canopy cover. Likewise, transients tended to walk in areas with reduced forest canopy cover, but selected areas farther away from roads. We recognize that inferred behavioral states are not well‐defined behaviors, so walking may have also included activities such as territorial marking, which is often associated with roads (Barja & List, 2014). If so, resident coyotes would be expected to walk roads whereas transients would not. Regardless, the findings that walking behaviors were negatively associated with increasing vegetation density and areas with more open forest canopy suggest that coyotes logically avoid denser vegetation that may hinder movements, instead of walking through areas with more open forest canopy (i.e., open areas in general) that allow efficient movements.

Prey switching is a common foraging behavior exhibited by generalist predators (Murdoch, 1969; Oaten & Murdoch, 1975), and although coyotes are known to exhibit substantive plasticity in prey selection (Bekoff, 1977), they also can be selective foragers when the availability of prey dictate such (Prugh, 2005; Windberg & Mitchell, 1990). Coyotes use varying hunting strategies relative to size and distribution of prey species within their territories (Gese & Grothe, 1995; Wells & Bekoff, 1982), and white‐tailed deer are an important prey source for coyotes in the Southeast (Chitwood et al., 2015; Shuman et al., 2017; Ward et al., 2018). Deer typically flee in response to coyotes approaching (Lingle, 2001), so coyotes attempting to prey on deer may be using a hunting strategy (i.e., chasing) that we could have inferred, at least partially, to be walking behaviors. Likewise, previous authors have noted that coyote use of deer was positively associated with areas of reduced vegetation density in their home ranges, presumably because of improved vision and olfaction (Cherry et al., 2016; Ward et al., 2018).

Coyotes should spend more time foraging in profitable areas and engage in area‐restricted search behavior in areas of greater prey density (Fauchald, 1999; Fauchald & Tveraa, 2003; Kareiva & Odell, 1987). We found that resident coyotes tended to forage closer to riparian areas and in areas with denser vegetation, whereas transients foraged closer to forest edges in areas with reduced canopy cover farther away from roads. Because coyotes are coursing predators (Bleich, 1999; Pierce et al., 2000), it is also plausible that the inferred behavior of foraging (area‐restricted searching) also included situations where coyotes were handling larger prey after making kills. Prey selection of coyotes during reproductive periods often focuses on small mammals, lagomorphs, soft mast, and neonate ungulates, which are readily available in early successional habitats with abundant ground‐level vegetation (Cherry et al., 2016; Patterson & Messier, 2000; Ward et al., 2018). Coyotes were historically present in the central and western regions of North America (Nowak, 2002), where they were adapted to foraging on relatively small prey species in open, early successional plant communities (Bekoff & Gese, 2003; Mills & Knowlton, 1991). However, coyotes throughout the southeastern United States have larger body sizes coupled with shorter ears and tails, likely adaptations to facilitate effective foraging in areas with denser vegetation and larger prey, suggesting that the need to exhibit plasticity in habitat and prey selection has facilitated morphological and behavioral adaptations in southeastern coyotes (Hinton et al., 2019).

## CONFLICT OF INTEREST

The authors declare no conflicts of interest exist.

## AUTHOR CONTRIBUTIONS

**Michael J. Chamberlain:** Conceptualization (lead); Data curation (supporting); Formal analysis (supporting); Funding acquisition (lead); Project administration (lead); Writing‐original draft (lead). **Bradley S. Cohen:** Formal analysis (supporting); Methodology (supporting); Writing‐original draft (supporting). **Patrick H. Wightman:** Data curation (supporting); Formal analysis (supporting); Methodology (supporting); Writing‐original draft (supporting). **Emily Rushton:** Project administration (supporting); Resources (supporting). **Joseph W. Hinton:** Conceptualization (supporting); Data curation (supporting); Formal analysis (supporting); Methodology (supporting); Writing‐original draft (supporting).

## Supporting information

Appendix S1

## Data Availability

The data files for behavioral change‐point analysis, space use, and movements, and the landcover data used in all analyses are available upon request or can be accessed on Dryad (https://doi.org/10.5061/dryad.fqz612jsd).
