# Supplementary material for: Fine‐scale movements and behaviors of coyotes (Canis latrans) during their reproductive period
Source: Ecol Evol. 2021 Jun 15;11(14):9575–88. doi: 10.1002/ece3.7777 (PMC8293769; doi:10.1002/ece3.7777)
Supplement: Supplementary file 1 — Appendix S1 [file ECE3-11-9575-s001.docx]

APPENDIX S1


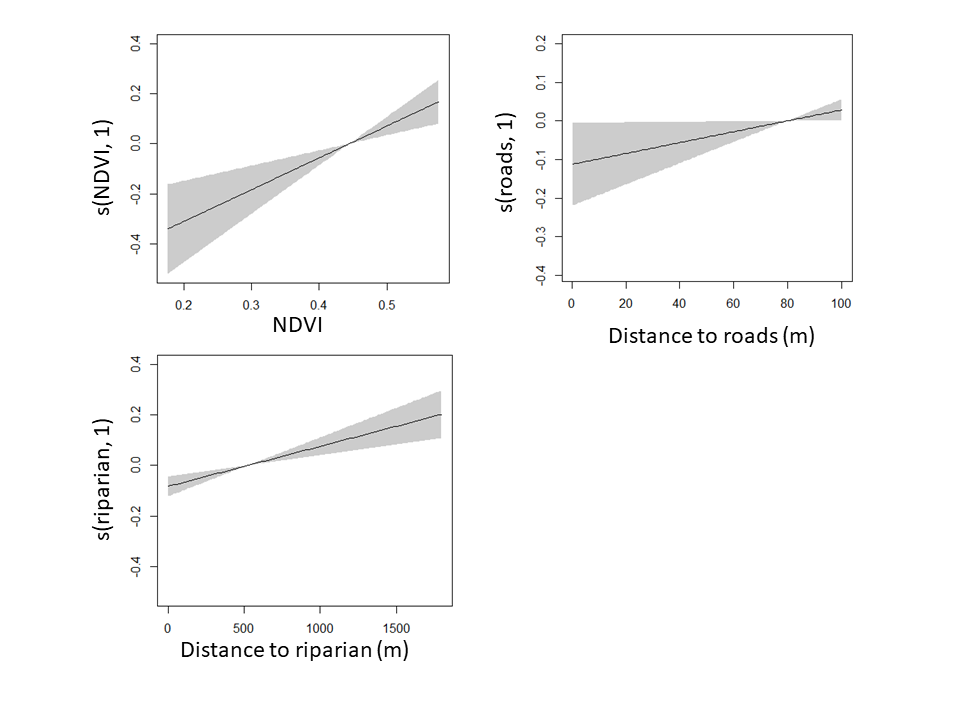


Predicted effects of vegetation density (NDVI), distance to roads, and distance to riparian areas on resting behaviors by resident coyotes on Cedar Creek and B. F. Grant Wildlife Management Areas and surrounding private lands in Georgia during 2018-2019.


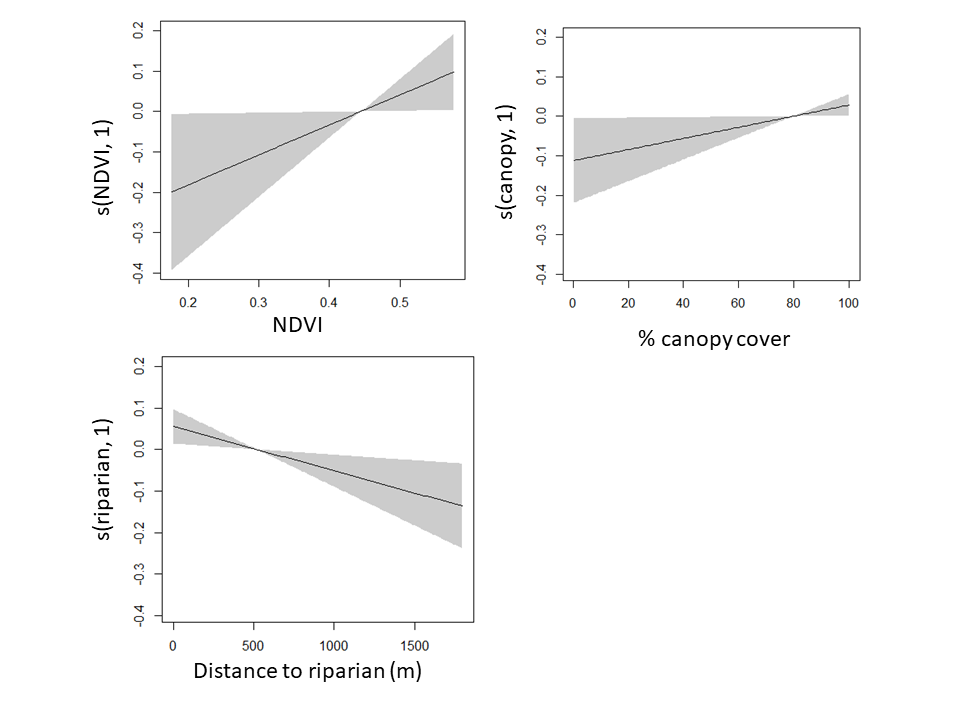


Predicted effects of vegetation density (NDVI), distance to riparian areas, and percentage (%) canopy cover on foraging behaviors by resident coyotes on Cedar Creek and B. F. Grant Wildlife Management Areas and surrounding private lands in Georgia during 2018-2019.


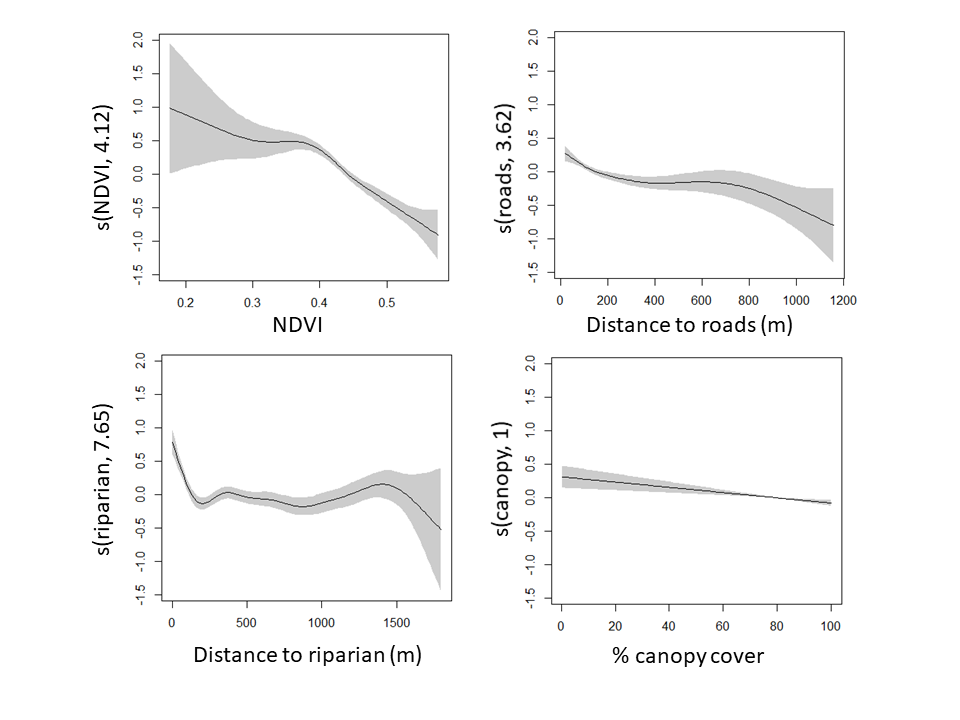


Predicted effects of vegetation density (NDVI), distance to roads, distance to riparian areas, and percentage (%) canopy cover on walking behaviors by resident coyotes on Cedar Creek and B. F. Grant Wildlife Management Areas and surrounding private lands in Georgia during 2018-2019.


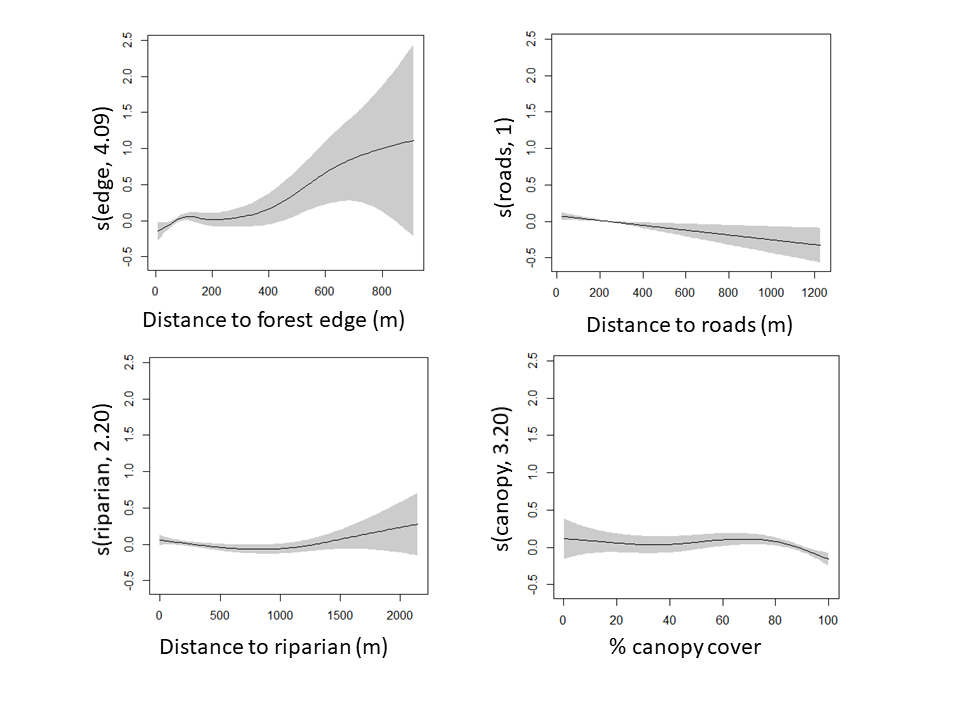


Predicted effects of distance to forest edge, distance to roads, distance to riparian areas, and percentage (%) canopy cover on resting behaviors by transient coyotes on Cedar Creek and B. F. Grant Wildlife Management Areas and surrounding private lands in Georgia during 2018-2019.


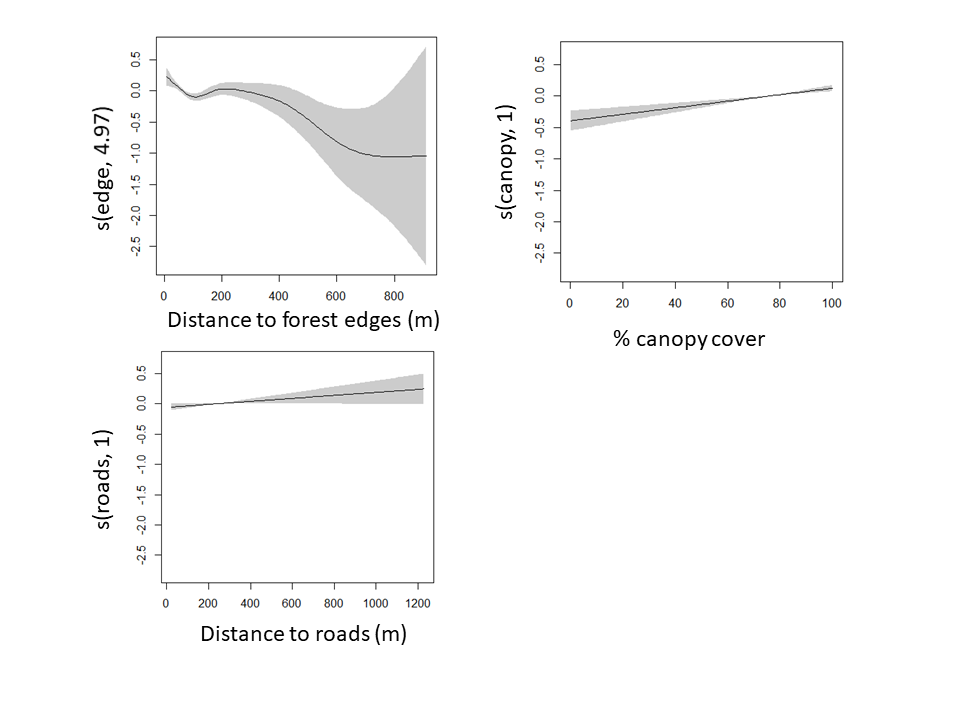


Predicted effects of distance to forest edge, distance to roads, and percentage (%) canopy cover on foraging behaviors by transient coyotes on Cedar Creek and B. F. Grant Wildlife Management Areas and surrounding private lands in Georgia during 2018-2019.


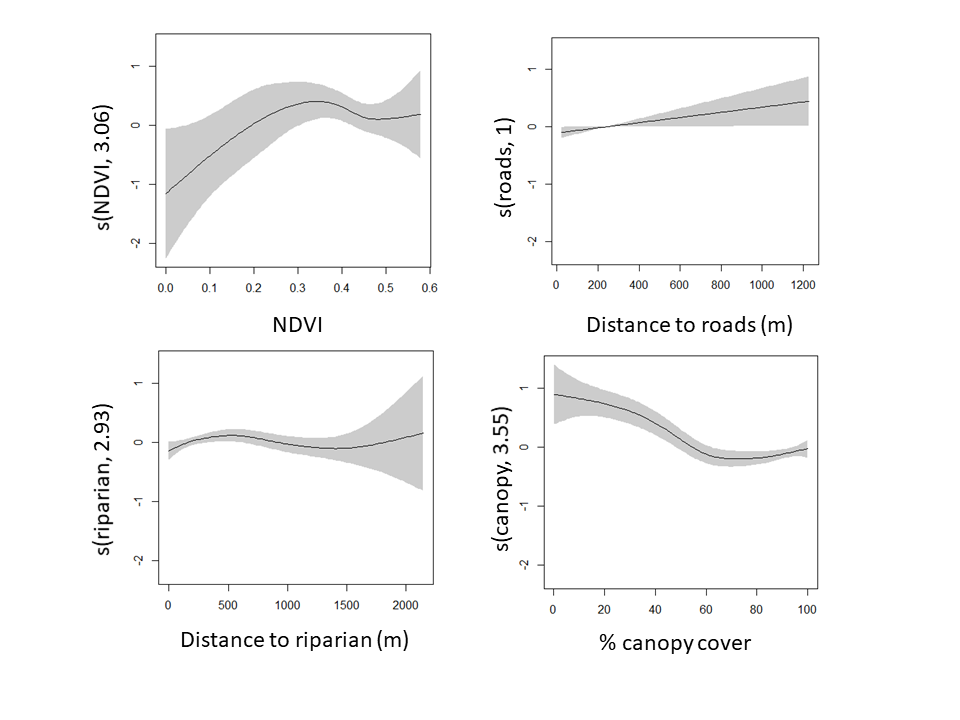


Predicted effects of vegetation density (NDVI), distance to roads, distance to riparian areas, and percentage (%) canopy cover on walking behaviors by transient coyotes on Cedar Creek and B. F. Grant Wildlife Management Areas and surrounding private lands in Georgia during 2018-2019.
